# Foreign allometric exponents adequately normalize isokinetic knee extension strength to identify muscle weakness and mobility limitation in Portuguese older adults: a cross-sectional study

**DOI:** 10.1186/s12877-022-03413-9

**Published:** 2022-09-16

**Authors:** Dalmo Roberto Lopes Machado, Pedro Pugliesi Abdalla, Lucimere Bohn, Gareth Stratton, Jorge Mota

**Affiliations:** 1 College of Nursing at Ribeirão Preto, University of São Paulo, Ribeirão Preto, Brazil; 2 School of Physical Education and Sport of Ribeirão Preto, University of São Paulo, Ribeirão Preto, Brazil; 3 Faculty of Sports, University of Porto, Porto, Portugal; 4 Faculty of Sports, Research Center in Physical Activity, Health and Leisure, University of Porto, Porto, Portugal; 5 Laboratory for Integrative, Translational Research in Population Health (ITR), Porto, Portugal; 6 Faculty of Psychology, Education and Sport, Lusófona University of Porto, Porto, Portugal; 7 Technology, Exercise and Medicine Research Centre, Swamsea University of, Applied Sport, Swansea, UK

**Keywords:** Functional performance, Geriatric assessment, Geriatric medicine, Health science, Longevity, Physical function, Public health, Scaling, Sports medicine

## Abstract

**Background:**

Identifying muscle weakness is challenging, because the reduction of strength with aging does not depend only on sarcopenia, but also on sensorimotor deficits. Nevertheless, this identification is improved by adjusting muscle strength allometrically, by removing the influence of body size. However, the effectiveness of foreign models to normalize these (dys)functionalities is not yet tested. This study aimed to compare and apply foreign allometric exponents for normalizing isokinetic knee extension strength in Portuguese older adults to identify muscle weakness/mobility limitation. Additionally, to attest any populational difference, data of these people and Brazilian older adults were compared

**Methods:**

This is a cross-sectional study encompassing 226 Portuguese (*n* = 132) and Brazilian (*n* = 94) older adults. Mobility limitation (six-minute walk test, at lowest quartile), lower limb strength (knee extension isokinetic strength at 60º/s), and body dimensions measures were taken. Foreign allometric exponents (^b^) were used to normalize Portuguese strength (strength/body-size variables^b^). Non-normalized and normalized strength were compared (ROC) to generate the most accurate cut-point for identifying muscle weakness/mobility limitation.

**Results:**

Older Portuguese men and women had better mobility than their Brazilian counterparts. Older Portuguese women had superior muscle strength to Brazilian women. Normalization from 11 foreign models removed the influence of body size on muscle strength, with a negligible correlation (r ≤ 0.30). In contrast to the non-normalized strength, the normalized strength cut-off points were sufficiently accurate (AUC ≥ 0.70) to avoid identifying false-negative cases of weakness/mobility limitation.

**Conclusions:**

Portuguese older women were stronger and had superior functional capacity compared to Brazilian ones. Normalized foreign models improved the accuracy in identifying muscle weakness/mobility limitation in Portuguese older adults. The isokinetic knee extension muscle strength normalized, even using foreign allometric exponents, should be better than no adjustment.

## Introduction

Muscle weakness occurs with aging and predicts clinically relevant health outcomes in older adults [1–3] such as disability (e.g. mobility limitation) [4] and multimorbidity in predicting all-cause mortality in older adults [5]. Therefore diagnosing muscle weakness, using muscle strength assessment [6], is important to identify dynapenia [7], frailty [8], and sarcopenia [6]. In older adults, muscle strength declines three times more quickly than skeletal muscle mass [9], with leg strength declining earlier than upper limbs muscle strength [10]. Strength deficits do not result from reduced muscle mass alone, but are also caused by sensorimotor deficits, and the expression of muscle strength is a consequence of sensorimotor abilities [11, 12]. However, measuring sensorimotor abilities/deficits is not an easy task, and strength can be measured in a more simple way [11, 12], which presents association with clinically relevant health-related outcomes for older adults [5].

While weak isokinetic knee extension is the best predictor of mobility limitation [13, 14], current isokinetic strength indexes to identify mobility limitation are based on absolute strength results [15–19] or when normalized by body mass using ratio standards [20]. However, the “muscle weakness” phenotype may be incorrectly applied to older adults who have a lighter body mass and shorter stature using absolute cutoff points [21–23], even if they sustain their mobility [14]. This false-positive diagnosis of muscle weakness can lead to the unnecessary use of health resources [24]. These misclassifications of the condition ´mobility limitation´ result from the nonlinear relationship between muscle strength and body-size variables [21–23]. Because allometric scaling contemplates power and sensitivity in this nonlinear relationship with the allometric exponent (^b^), it overcomes the aforementioned constraints [14, 21–23]. The ^b^ scale one outcome variable (Y) to another variable (X), but free of the undue influence of the X. Y scaled variable (Y × X^−b^) is a result of Y without the independent effects of the scaling variable (X). A nonlinear relationship is confirmed when ^b^ is between 0.00 and 0.99, although when ^b^ is ≥ 1.00 a linear relationship is characterized [25].

The nonlinear relationship between isokinetic leg extension strength and body mass in older adults has been reported [13, 26, 27], with ^b^ varying between 0.37 to 0.74. Indeed, scaling isokinetic knee extension strength by body size removes the effect of body size on muscle strength [13]. Furthermore, where muscle strength was allometrically adjusted, the accuracy in identifying muscle weakness and mobility limitation was improved compared to non-normalized values in older adults. Brazilian [13] and North American [26, 27] allometric exponents are already available to normalize isokinetic knee extension strength, however, their validity has not been assessed in other populations.

The study aims are 1) to compare the mobility capacity and muscle strength of older adults in Portugal and Brazil and 2) to identify muscle weakness/mobility limitation in older Portuguese adults using allometric exponents to normalize isokinetic knee extension strength. We hypothesize that the muscle strength normalized using allometric exponents demonstrates that muscle strength changes independent of body size. We recommend that these should be used to improve the accuracy of identifying muscle weakness/mobility limitation.

## Methods

### Study design and population

This is a cross-sectional study with data of two samples, one from Brazil (measured at University Hospital of Ribeirao Preto School of Medicine, University of Sao Paulo, Brazil (HC-FMRP-USP) and another from Portugal (measured in Faculty of Sports, University of Porto, Portugal [FADEUP]). Both studies obeyed the Helsinki Declaration and were approved by their respective institutional review board. Older adults were voluntarily recruited, and all of them have been assigned informed consent. This manuscript still followed the guidelines from The Strengthening the Reporting of Observational Studies in Epidemiology (STROBE) conference list.

Both Brazilian and Portuguese samples consisted of community-dwelling older adults (> 60 years old). The Brazilian sample was recruited from social projects on behalf of older adults of USP and from health community services of the same institution and city. The Portuguese sample was recruited through advertisements in newspapers from the Porto metropolitan area. Inclusion criteria for the Brazilian sample were walking independently, absence of limitations to execute all procedures, no acute infection, cancer diagnosis, hip or knee prostheses, an unstable cardiovascular condition, stroke sequelae, cancer, weight loss > 3 kg in the last three months or cognitive impairment. The Portuguese sample was aged 60–85 years, of community-dwelling status, did not use bone-acting drugs, vitamin D, and calcium, or have a significant sensory/cognitive impairment or medical.

### Procedures

A multidisciplinary health team (Brazilian sample) and researchers of the Faculty of Sports (Portuguese sample) performed data collection.

### Cognition assessment

The validated Mini-Mental State Examination (MMSE) was used to assess participants´ cognition status in the Brazilian sample and those who have MMSE ≤ 12 were excluded [28].

### The measure of body-size variables

Body-size variables were collected to compare the anthropometric profile of Portuguese and Brazilian older adults and to normalize their performance in muscle strength tests. The selection of these variables was based on those previously used to calculate body indexes [29], and involved the anthropometric measurements body mass [digital medical scales Filizola® (model Personal, MS, Brazil) for Brazilian sample; and Seca (GmbH, model 708, Germany) for Portuguese sample], height (using the stadiometer Sanny® Professional (model ES2020, Brazil) for Brazilian sample, and Seca 220 (Germany) for Portuguese sample], waist circumference [30] with a tape measure (both samples) and body composition by Dual Energy X-ray Absorptiometry (DXA; QDR 4500A, Hologic, Bedford, MA for both samples), as briefly detailed below.

### Body indexes

The body indexes derived from anthropometry were body mass index (BMI, kg/m^2^) [31], body mass*height [27], and human body surface area (SA, m^2^) [29]. Body indexes derived from body composition by DXA were lean soft tissue (LST), appendicular skeletal muscle mass (ASM), and fat-free mass (FFM) when fat mass was estimated from body mass difference.

### Mobility measurement

Muscle weakness cut-off points were based on poor mobility (lowest quartile of mobility performance) [32]. Mobility performance was verified using the six-minute walk test (6MWT) executed in a corridor 30-m length (Brazilian sample) and 45-m length (Portuguese sample). Along the corridor, cones were positioned at five-meter intervals to help researchers to identify the distance walked [33]. Participants were instructed to cover the longest distance and walk as fast as they could over the six-minute. Participants could slow down or interrupt their walking, and resume the test whenever desired. The total walked distance was recorded.

### Muscle strength measurements

The isokinetic knee extension peak torque at 60°/s of the right lower limb (PT) was recorded with the isokinetic dynamometer (Biodex System 4 Pro; Biodex, Shirley, NY in both samples). Detailed protocols have previously been published for Brazilian and Portuguese studies [34]. The major differences between protocols were in the warm-up.

The warm-up for the Brazilian sample consists of 10 submaximal repetitions at velocity 60º/s. The warm-up for the Portuguese sample consists of five minutes on a bicycle ergometer (Bike‐Max; Tectrix, Irvine, CA) at 45–60 W. PT was obtained with maximal efforts, consisting of five repetitions at 60º/s for the Brazilian sample (executed three minutes after warm-up) and three repetitions at 60º/s for the Portuguese sample (but executed two minutes after five repetitions in a maximal effort at 180º/s). The PT in Newton-meter (Nm) was considered being the highest value found from all repetitions executed.

### Muscle strength normalization procedures (allometric scaling)

PT was considered in two different ways: 1) absolute (non-normalized) and 2) allometrically adjusted (muscle strength/body-size variables^**b**^). Allometric exponents (^**b**^) were considered from the literature, as described in Table 1.

**Table 1:** Brazilian and North American allometric exponents (^b^) proposed in previous studies to normalize isokinetic knee extension peak torque at 60°/s (PT)

Authors	Nationality	Normalized PT for body-size variable
Abdalla et al., 2021 [13]	São Paulo, Brazil	/height^**3.27**^
		/(body mass ^*^height)^**0.43**^
		/SA^**0.83**^
		/left leg LST^**0.43**^
		/right leg LST^**0.48**^
		/legs LST^**0.47**^
Davies and Dalsky (1997) [26]	New Mexico, USA	/body mass^**0.67**^
		/body mass^**0.72**^
		/body mass^**0.74**^
Segal et al., (2008) [27]	Iowa, USA	/body mass ^*^height^**0.97**^

^*^ one example of normalization: PT/body mass*height^0.97^

To verify whether normalization (PT/body-size variable^**b**^) removed the influence of body size on muscle strength, the correlation between normalized muscle strength and body-size variables (body mass, height, and body-size used) should be negligible (r ≤ 0.30) [35].

### Statistical analysis

Descriptive statistics (mean, 95% CI and standard deviation) and independent samples t-tests examined mobility capacity (mobility and muscle strength) differences between nationalities.

### The proposition of cut-off points for muscle weakness

Allometrically scaled and absolute muscle strength areas under the curve were quantified using ROC analyses. The Youden index selected the most appropriate cut-off points with the best relationship between sensitivity and specificity for the primary outcome (poor mobility). Poor mobility was chosen as reference variable to propose weakness cut-off points because it is a relevant health-related outcome for older adults [36] and it was considered in other studies to propose cut-off points of muscle strength to identify sarcopenia [14, 37].

For each body-size variable and sex, the ROC curves of non-normalized (continuous line) and normalized muscle strength (dashed lines) were compared to each other to decide the best cut-off point.

Analyzes were carried out using the SPSS 25.0 statistical package, and the ROC curves and Youden index with NCSS 2021 with a previously established level of significance (α = 5%).

## Results

**Fig. 1:**
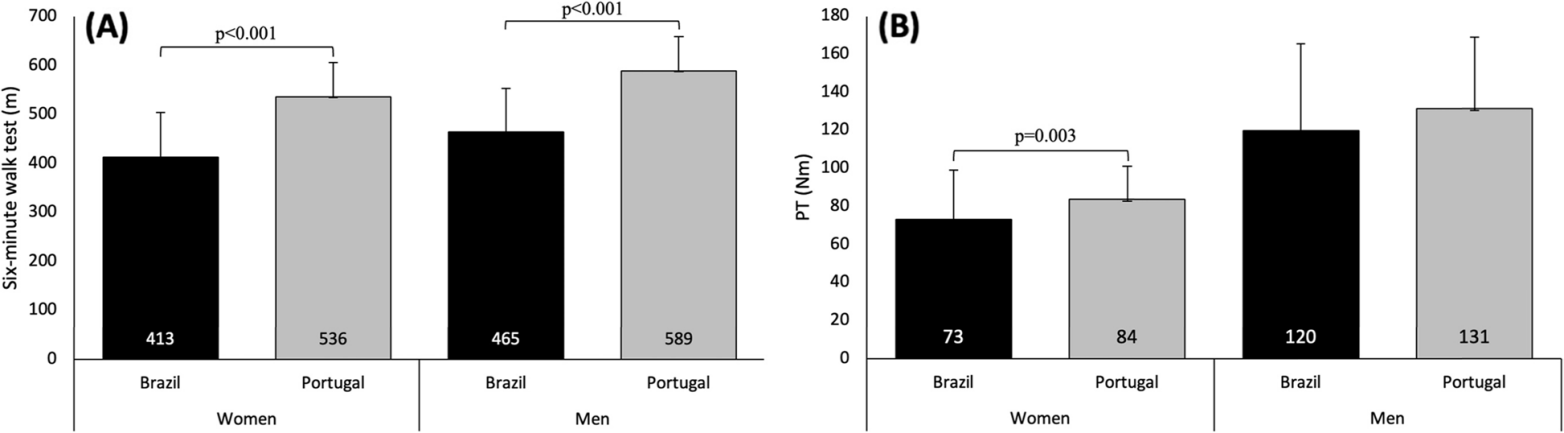
Comparison of functional capacity (**A**) and isokinetic knee extension peak torque at 60º/s (PT; **B**) among Brazilian and Portuguese older adults

The Brazilian sample encompassed 94 older adults (69 women, 69.1%) and the Portuguese one, 132 (94 women, 71.2% women). Sample characterization according to nationality and sex is shown in Table 2. Between nationalities comparisons according to sex, show that Portuguese men had a higher body mass, BMI, and SA than their Brazilian counterparts, while the Brazilian women had higher stature than Portuguese women. For both sexes, Portuguese participants presented with higher ASM, ASM/height^2^, and mobility in 6MWT (Fig. 1) than Brazilians. Differences for muscle strength (PT) were noted for women (Fig. 1) and these differences were preserved after normalization for almost all body size variables (Table 1). Twenty-four Portuguese women (25.5%) and twenty-eight Portuguese men (26.3%) had poor mobility performance (6MWT < lowest quartile).

**Table 2:** Descriptive and comparative analysis of community-dwelling older adults in Brazil and Portugal

	**Unit**	**Women**									**Men**								
		**Brazil (*n* = 65)**				**Portugal (*n* = 94)**				***p***	**Brazil (*n* = 29)**				**Portugal (*n* = 38)**				***p***
		**M**	**95% CI**		**SD**	**M**	**95% CI**		**SD**		**M**	**95% CI**		**SD**	**M**	**95% CI**		**SD**	
			**LL**	**UL**			**LL**	**UL**				**LL**	**UL**			**LL**	**UL**		
Age	Years	69.7	68.2	71.2	6.1	68.5	67.4	69.6	5.3	0.197	71.2	68.5	73.9	7.1	69.4	67.5	71.3	5.9	0.267
Body mass	kg	66.9	64.0	69.8	11.6	65.8	63.7	67.9	10.1	0.519	73	67.7	78.3	13.9	81	77.5	84.4	10.5	0.009
Height	m	1.6	1.5	1.6	0.1	1.5	1.5	1.5	0.1	< 0.001	1.7	1.6	1.7	0.1	1.7	1.7	1.7	0.1	0.627
BMI	kg/m^2^	27.4	26.3	28.5	4.4	28.3	27.5	29.1	4.1	0.194	25.7	24.3	27.2	3.8	28.9	27.9	30.0	3.3	< 0.001
Waist circumference	cm	86.5	84.0	89.0	10	89.7	87.4	92.0	8.6	0.068	92.1	87.8	96.5	11.4	91.1	88.5	93.7	5	0.738
SA	m^2^	1.7	1.7	1.8	0.2	1.7	1.7	1.7	0.1	0.212	1.9	1.8	1.9	0.2	2.0	1.9	2.0	0.1	0.024
ASM (kg)	kg	14.5	13.9	15.1	2.5	15.3	14.8	15.9	2.1	0.048	20.9	19.3	22.5	4.2	23.5	22.1	24.9	3.3	0.016
ASM/height^2^	kg/m^2^	5.95	5.7	6.2	0.95	6.58	6.4	6.8	0.77	< 0.001	7.34	7.0	7.7	0.99	8.45	8.0	8.9	1.01	< 0.001
Six-minute walk test (6MWT)	m	412.7	389.9	435.5	92	536	521.6	550.4	70.2	< 0.001	464.7	431.1	498.3	88.3	588.8	565.7	611.9	70.2	< 0.001
Non-normalized PT	Nm	73.2	66.8	79.6	25.9	83.8	80.2	87.5	17.4	0.003	119.8	102.4	137.2	45.6	131.4	118.9	143.9	37.5	0.262
Normalized PT																			
/body mass^0.74 (DAVIES; DALSKY, 1997)^	Nm/kg	3.3	3.0	3.6	1.1	3.9	3.7	4.0	0.9	0.001	5.0	4.4	5.6	1.6	5.1	4.7	5.5	1.3	0.776
/body mass^0.72 (DAVIES; DALSKY, 1997)^	Nm/kg	3.6	3.3	3.9	1.2	4.2	4.0	4.4	0.9	0.001	5.4	4.8	6.1	1.8	5.5	5.1	6.0	1.4	0.754
/body mass^0.67 (DAVIES; DALSKY, 1997)^	Nm/kg	4.4	4.0	4.8	1.5	5.2	4.9	5.4	1.1	0.001	6.7	5.9	7.6	2.2	6.9	6.3	7.5	1.7	0.702
/(body mass*height)^0.97 (SEGAL et al., 2008)^	Nm/kg*m	0.8	0.7	0.9	0.3	1	0.9	1.0	0.2	< 0.001	1.1	1.0	1.2	0.3	1.1	1.0	1.2	0.3	0.736
/height^3.27 (ABDALLA et al., 2021)^	Nm/m	16.9	15.6	18.3	5.4	21.2	20.3	22.1	4.3	< 0.001	21.5	19.0	24.0	6.6	24.3	22.2	26.4	6.3	0.082
/(body mass*height)^0.43 (ABDALLA et al., 2021)^	Nm/kg*m	9.7	8.9	10.5	3.3	11.7	11.2	12.2	2.4	< 0.001	14.7	12.8	16.5	4.9	15.9	14.5	17.2	4.1	0.283
/SA^0.83 (ABDALLA et al., 2021)^	Nm/m^2^	42.6	39.0	46.2	14.4	54.7	52.3	57.0	11.3	< 0.001	63.8	55.9	71.8	20.9	75	68.6	81.5	19.3	0.028
/left leg LST^0.43 (ABDALLA et al., 2021)^	Nm/g	1.8	1.7	2.0	0.6	2	1.9	2.1	0.4	0.085	2.6	2.3	2.9	0.9	2.8	2.4	3.1	0.7	0.507
/right leg LST^0.48 (ABDALLA et al., 2021)^	Nm/g	1.1	1.1	1.2	0.4	1.3	1.2	1.4	0.3	0.011	1.6	1.4	1.8	0.5	1.8	1.5	2.0	0.4	0.215
/legs LST^0.47 (ABDALLA et al., 2021)^	Nm/g	0.9	0.8	1.0	0.3	1.0	1.0	1.1	0.2	0.002	1.2	1.1	1.4	0.4	1.4	1.2	1.6	0.3	0.134

*Note*: *M* mean, *CI* confidence interval, *LL* lower limit, *UL* upper limit, *SD* standard deviation, *SA* human body surface area, *LST* lean soft tissue, *ASM* appendicular skeletal muscle mass, *Nm* Newtons-meter

**Fig. 2:**
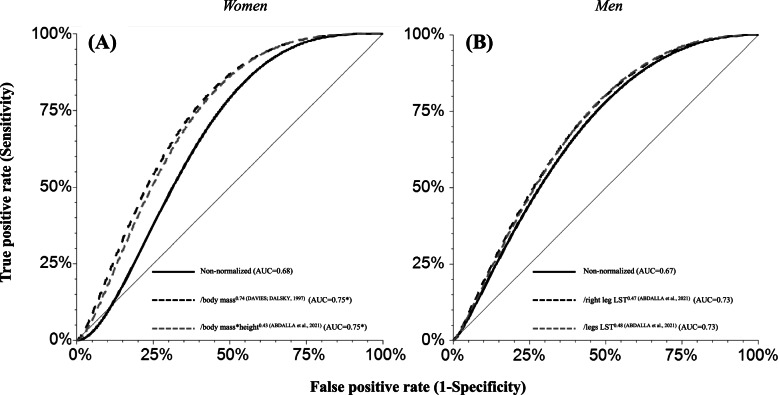
Accuracy comparison of absolute (non-normalized) and normalized isokinetic (better two) knee extension peak torque at 60º/s (PT) with international and Brazilian allometric exponents to identify poor mobility performance (lowest quartile of six-minute walk test) in Portuguese older adults’ women (**A**) and men (**B**). *p < 0.001 (greater than the AUC of non-normalized)

The sex-specific cut-off points proposed for PT (non-normalized and allometrically adjusted) to identify muscle weakness are presented in Table 3. Table 3 also shows correlations between muscle strength and body size (body mass, height, and body-size variable used in normalization). When PT was normalized, some derived cut-off points presented adequate accuracy (AUC ≥ 0.70) to identify muscle weakness in both sexes, however only one [/(body mass*height)^0.97 (SEGAL et al., 2008)^] was dependent on body size (with r > 0.30). Non-normalized PT cut-off points were not adequate for both men and women as they did not present sufficient accuracy (AUC ≥ 0.70) to identify muscle weakness [38]. To arrive at the best cut-point to identify muscle weakness, we compared that had higher accuracy and negligible correlation (r ≤ 0.30) with body size variables (Fig. 2). Only after normalizing muscle strength, did the AUC result in acceptable values for identifying mobility limitation (≥ 0.70; Fig. 2).

**Table 3:** Application of international and Brazilian allometric exponents in Portuguese community-dwelling older adults to normalize isokinetic knee extension peak torque at 60º/s (PT), their accuracy, and cut-off points to identify poor functional performance (lowest quartile of six-minute walk test)

PT	Unit	Portuguese women				Portuguese men				Correlation (r) with body size		
		**AUC**	**Cut-off point ( ≤)**	**sens (%)**	**spec (%)**	**AUC**	**Cut-off point ( ≤)**	**sens (%)**	**spec (%)**	**Body mass**	**Height**	**Normalization**
Non-normalized	Nm	0.68	87.4	86	51	0.67	132.4	90	48	0.28 ^*^	0.29 ^*^	
/body mass^0.74 (DAVIES; DALSKY, 1997)^	Nm/kg	0.75	4.10	100	47	0.69	4.46	70	74	-0.19	0.08	
/body mass^0.72 (DAVIES; DALSKY, 1997)^	Nm/kg	0.75	4.46	100	47	0.69	4.89	70	74	-0.18	0.08	
/body mass^0.67 (DAVIES; DALSKY, 1997)^	Nm/kg	0.74	5.51	100	46	0.68	6.15	70	74	-0.15	0.10	
/(body mass ^*^height)^0.97 (SEGAL et al., 2008)^	Nm/kg ^*^m	0.78	1.00	90	60	0.71	0.98	70	74	-0.41 ^*^	-0.15	-0.38^*^
/height^3.27 (ABDALLA et al., 2021)^	Nm/m	0.74	21.0	86	63	0.69	18.8	60	85	-0.01	-0.26 ^*^	
/(body mass ^*^height)^0.43 (ABDALLA et al., 2021)^	Nm/kg^*^m	0.75	12.5	100	49	0.69	13.2	60	78	-0.03	0.11	0.02
/SA^0.83 (ABDALLA et al., 2021)^	Nm/m^2^	0.74	58.7	100	47	0.69	62.5	60	78	-0.02	0.12	0.01
/left leg LST^0.43 (ABDALLA et al., 2021)^	Nm/g	0.69	2.14	93	49	0.72	2.83	100	50	0.07	0.15	0.11
/right leg LST^0.48 (ABDALLA et al., 2021)^	Nm/g	0.70	1.40	93	46	0.73	1.67	80	75	0.04	0.14	0.10
/legs LST^0.47 (ABDALLA et al., 2021)^	Nm/g	0.70	1.10	93	46	0.73	1.31	80	75	0.05	0.14	0.10

^*^ *p* < 0.05: statistically significant correlation

*Note. AUC* area under the curve; sens = sensibility; specificity; *Nm* Newtons meter, *SA* human body surface estimated by Bailey and Briars equation, *LST* lean soft tissue

## Discussion

The study aims were to compare the functional capacity and muscle strength of older adults in Portugal and Brazil and identify mobility limitations in older Portuguese adults using allometric exponents to normalize isokinetic knee extension strength.

Cut-points were tested to identify muscle weakness in older Portuguese adults based on lower limbs muscle strength normalized with allometric exponents from other countries (from Brazil and North America). The non-normalized cut-off points for lower limbs strength did not present sufficient accuracy (AUC ≥ 0.70) to identify muscle weakness and were not adequate for men or women. We intended to test international allometric exponents to older Portuguese adults. After normalizing lower limb strength with allometric exponents there were eleven accurate models (women = 8; men = 3) to identify false-negative cases of muscle weakness. In addition, after normalization, the association with body size was reduced for non-significant levels, except for “(body mass*height)^0.97 (SEGAL et al., 2008)^” and “height^3.27 (ABDALLA et al., 2021)^”. Normalized models of both sexes, without correlation with body size, isolate the natural interdependence between muscle strength and body size. The comparison of mobility capacity according to birthplace shows that older Portuguese adults have better mobility (both sexes) and superior muscle strength against older Brazilian women.

We assessed muscle weakness cut-points for PT allometrically adjusted with international allometric exponents in older Portuguese adults. In the literature, there are also muscle weakness cut-points for PT linearly normalized (ratio standard) by body mass [20]. However, that study did not compare the accuracy of allometrically adjusted with non-normalized muscle strength to identify mobility limitation/muscle weakness. Furthermore, the authors did not explore the natural interdependence between muscle strength and body size. When PT was linearly normalized by body mass, this variable presented correlation (r ≥ 0.30) with body size [13], which prevents recommending its use.

The normalization of PT with North American allometric exponents did not result in acceptable accuracy to identify muscle weakness in Portuguese men. There were no considerable differences reported in the literature in the six-minute walk test between Portuguese and North American older men [39]. Furthermore, differences for some anthropometric variables of North American men (29.6 ± 4.6 kg/m^2^; 80.8 ± 10.2 kg and 174.4 ± 7.0 cm [26, 27]) did not demonstrate considerable differences compared to Portuguese men (Δ of + 0.2 kg; and -0.7 kg/m^2^), but a not-negligible difference for height (Δ of -7.0 cm) was found and can somehow explain the lack of accuracy of the North American allometric exponents applied for Portuguese samples. Therefore, in addition to the anthropometric difference influencing accuracy, other factors still need to be studied and may require test across countries the necessity for specific allometric exponents. There are differences between countries of different incomes (e.g., Portugal vs the USA) regarding biological, early growth, nutrition, and genetic factors (ethnicity differences) that impact muscle strength.

Previous studies have proposed allometric exponents to normalize PT by body mass, height, body mass*height, SA and DXA derived LST [13, 26, 27]. All allometric exponents were tested in our sample and most of them were accurate enough to identify muscle weakness. Although the variables “(body mass*height)^0.97 (SEGAL et al., 2008)^” and “height^3.27 (ABDALLA et al., 2021)^” were accurate enough to identify muscle weakness, they were correlated with body size (Table 3). The linear relationship between height and strength may explain this association. A linear relationship between two variables occurs when ^b^ ≥ 1.00), where ^b^ in the literature for height and muscle strength varies between 1.46 and 3.27 [13, 22, 40]. A non-linear relationship between two variables occurs when ^b^ < 1.00, e.g., in a previous study the variable “body mass*height” shows ^b^ of 0.974 with muscle strength [27]. Despite a curvilinear (allometric) relationship being confirmed when ^b^ is between 0.00 and 0.99, the dependency of “PT/(body mass*height)^0.97 (SEGAL et al., 2008)^” with body size (r > 0.30) can be possibly explained by the confidence interval. The authors did not report the confidence interval, but certainly, it exceeds the unity (^b^ ≥ 1.00), featuring a linear relationship with body size, which justifies the interdependence between muscle strength and body size. Notwithstanding, when an allometric scaling (^b=0.43^; [13]) is used for body mass*height, independence of body size (r between -0.03 and 0.11; Table 3) was reached, demonstrating the usefulness of allometry.

Some strengths of our study are noteworthy. We tested muscle weakness cut-off points from the “gold standard” to assess lower limbs strength (isokinetic dynamometer). A considerable number of allometric exponents (*n* = 10) were tested in our study, expanding the normalization possibilities of knee extension strength performed in an isokinetic dynamometer. Our findings can be applied to identify muscle weakness in clinical practice for both sexes with sufficient accuracy (AUC > 0.70), independently of body size. Nonetheless, this study has limitations such as its cross-sectional design, which may underestimate the decline in individual muscle strength because of the natural aging process. Additionally, because the sample size was small and constituted mostly for women, the extrapolation of our findings to other populations must be with caution. Another limitation is that PT was only measured on the right lower limb. However, there are no differences between dominant and nondominant limbs regarding the PT at 60°/s speed among older adults [41].

The isokinetic dynamometer is expensive and generally only available in research rather than clinical settings. Even though, our idea to normalize muscle strength can be also applicable in clinical practice with widely available instruments in geriatric environments like manual dynamometers. For this, allometric exponents proposed to normalize performed handgrip strength need to be tested for Portuguese older adults. The assessment of older adults’ muscle strength and muscle weakness classification should be frequent in clinical practice, to avoid unnecessary expenditures from false-positive cases election. Future studies can test allometric exponents to normalize muscle strength for different ethnicity/races of older adults.

As an applied example to avoid false-positive diagnosis for muscle weakness, we hypothesize one older Portuguese man, with extreme lower values of height (1.53 m) and right leg LST_DXA_ (6700 g), who performed PT of 130.0 Nm. If considered our absolute cut-off point (≤ 132.4 Nm), this person has “muscle weakness” confirmed. However, when considering the normalized PT/(right leg LST^0.48^), the adjusted value (1.89 Nm/g) is above the cut-off point (1.67 Nm/g; Table 3). For older people with large body sizes, normalizing strength would also prevent muscle weakness false-negative diagnosis. Mistakenly classified cases of muscle weakness can impact the financial resources of the healthcare and older adults care systems.

## Conclusion

Community-dwelling Portuguese older adults are stronger (women) and have better mobility capacity (both sexes) compared to the Brazilian ones. Despite that, some foreign allometric exponents (Brazilian and North American) can be utilized to normalize the knee extension strength of these Portuguese older adults, when this normalization strategy improves the accuracy to identify muscle weakness/mobility limitation for both sexes. Normalizing muscle strength, even with foreign allometric exponents, is better than using it in an absolute form (non-normalized) to identify muscle weakness/mobility limitation, against cases of false-positive diagnosis.

## Data Availability

The datasets generated and/or analyzed during the current study are available in the figshare repository, https://doi.org/10.6084/m9.figshare.19401032.v1

## Abbreviations

6MWT: Six-minute walk test
ASM: Appendicular skeletal muscle mass
AUC: Area under the curve
^b^: Allometric exponent
BMI: Body mass index
CAPES: Coordenação de Aperfeiçoamento de Pessoal de Nível Superior
CI: Confidence interval
CIAFEL: Center for Research in Physical Activity, Health and Leisure
CNPq: Conselho Nacional de Desenvolvimento Científico e Tecnológico
DXA: Dual-energy X-ray absorptiometry
FADEUP: Faculty of Sport, University of Porto, Portugal
FCT: Fundação para a Ciência e a Tecnologia
FFM: Fat-free mass
HC-FMRP-USP: University Hospital of Ribeirao Preto School of Medicine, University of Sao Paulo, Brazil
LST: Lean soft tissue
MMSE: Mini-Mental State Examination
PT: Isokinetic knee extension peak torque at 60°/s of the right lower limb
SA: Human body surface area
STROBE: Strengthening the Reporting of OBservational Studies in Epidemiology
WHO: World Health Organization
